# Topological Phase and Quantum Anomalous Hall Effect in Ferromagnetic Transition-Metal Dichalcogenides Monolayer $1T-V{\mathrm{Se}}_{2}$

**DOI:** 10.3390/nano11081998

**Published:** 2021-08-04

**Authors:** Angus Huang, Chin-Hsuan Chen, Ching-Hao Chang, Horng-Tay Jeng

**Affiliations:** 1 Department of Physics, National Tsing Hua University, Hsinchu 30013, Taiwan; rabbit4a9@gmail.com (A.H.); barry810929@gmail.com (C.-H.C.); 2 Department of Physics, National Cheng Kung University, Tainan 70101, Taiwan; cutygo@gmail.com; 3 Physics Division, National Center for Theoretical Sciences, Hsinchu 30013, Taiwan; 4 Institute of Physics, Academia Sinica, Taipei 11529, Taiwan

**Keywords:** 2D materials, magnetic materials, quantum anomalous Hall effect, transition-metal dichalcogenides

## Abstract

Magnetic two-dimensional (2D) van der Waals materials have attracted tremendous attention because of their high potential in spintronics. In particular, the quantum anomalous Hall (QAH) effect in magnetic 2D layers shows a very promising prospect for hosting Majorana zero modes at the topologically protected edge states in proximity to superconductors. However, the QAH effect has not yet been experimentally realized in monolayer systems to date. In this work, we study the electronic structures and topological properties of the 2D ferromagnetic transition-metal dichalcogenides (TMD) monolayer $1T-V{\mathrm{Se}}_{2}$ by first-principles calculations with the Heyd–Scuseria–Ernzerhof (HSE) functional. We find that the spin-orbit coupling (SOC) opens a continuous band gap at the magnetic Weyl-like crossing point hosting the quantum anomalous Hall effect with Chern number C=2. Moreover, we demonstrate the topologically protected edge states and intrinsic (spin) Hall conductivity in this magnetic 2D TMD system. Our results indicate that $1T-V{\mathrm{Se}}_{2}$ monolayer serves as a stoichiometric quantum anomalous Hall material.

## 1. Introduction

Topological phase has been one of the main themes in solid-state physics and materials science in the past decade. Especially, topological materials have—in view of their robust topological surface or edge states—shown great promise in spintronics. Since the study of graphene, Haldane shows that the Landau level can be presented in graphene if including an external term [1] such as the spin-orbital coupling (SOC), which is now known as the quantum spin Hall effect or $Z_{2}$ topological insulators [2]. After that, different kinds of topological phase and band inversion induced by various sources have been proposed. The discovery of tuning topological phases, whether through spin-orbital coupling (HgTe quantum well state [3] and ${\mathrm{Bi}}_{2}{\mathrm{Te}}_{3}$ [4]), electron–phonon coupling (BiTeI [5]), ion doping (${\mathrm{WTe}}_{2}$ [6]), and external strain (${\mathrm{Bi}}_{2}{\mathrm{Se}}_{3}$ [7], HgSe monolayer [8]), has opened new routes to control phases and transport properties.

In particular, the quantum anomalous Hall (QAH) effect, which presents topological properties in time-reversal symmetry breaking systems, has been reported in ${\mathrm{Cr}}_{x}{\left({\mathrm{Bi}}_{y}{\mathrm{Sb}}_{1-y}\right)}_{2-x}{\mathrm{Te}}_{3}$ [9], $\mathrm{Mn}{\mathrm{Bi}}_{2}{\mathrm{Te}}_{4}$ [10,11], and $\mathrm{Tb}{\mathrm{Mn}}_{6}{\mathrm{Sn}}_{6}$ [12]. Moreover, Majorana fermion mode has been demonstrated experimentally in heterostructures with quantum anomalous Hall materials adjacent to superconductors through proximity effects [13]. However, the QAH effect, the topological phase with magnetism, has not been realized experimentally in monolayer materials by far.

Novel magnetic two-dimensional (2D) materials, another widely studied class of layered materials bounded by van der Waals force, have attracted increasing attention in recent years owing to the great potential for spintronic applications. A number of monolayer magnetic materials, such as $V{\mathrm{Se}}_{2}$ [14,15], ${\mathrm{CrI}}_{3}$ [16], ${\mathrm{CrGeTe}}_{3}$[17], and ${\mathrm{Fe}}_{3}{\mathrm{GeTe}}_{2}$ [18] have been realized in experiments. Particularly, $1T-V{\mathrm{Se}}_{2}$ presents ferromagnetic properties with the Curie temperature $T_{\mathrm{Curie}}=470\;K$ [14] above room temperature (RT). $1T-V{\mathrm{Se}}_{2}$ has also been shown to exhibit the charge density wave (CDW) phase below 130 K [14,15,19]. The wide temperature range for the ferromagnetic phase from 130 K up to RT achieves $1T-V{\mathrm{Se}}_{2}$ a high potential candidate for fulfilling the QAH effect in a single TMD layer. It is, thus, important to study the topological properties and Hall conductivity in monolayer $1T-V{\mathrm{Se}}_{2}$ for it can open up a new route to spintronics, QAH, and even the Majorana physics.

In this work, we study the topological properties, Hall conductivity, and spin Hall conductivity in magnetic TMD monolayer $1T-V{\mathrm{Se}}_{2}$ via first-principle calculations based on density functional theory (DFT) with the hybrid Heyd–Scuseria–Ernzerhof (HSE) exchange-correlation functional. We show that magnetic monolayer $1T-V{\mathrm{Se}}_{2}$ exhibits a continuous band gap opened at the Weyl-like crossing point due to the spin-orbit coupling (SOC), leading to the topological phase with Chern number C=2. We also demonstrate the quantum anomalous Hall (QAH) effect given from the topologically protected edge states. Finally, we present our calculated Hall conductivity and spin Hall conductivity for future experiments to examine such transport properties in monolayer $1T-V{\mathrm{Se}}_{2}$.

## 2. Method

The first-principles calculations of monolayer $1T-V{\mathrm{Se}}_{2}$ are performed using the full-potential projected augmented wave method as implemented in the Vienna Ab-initio Simulation Package (VASP) [20,21] based on the density functional theory (DFT) with the Perdew–Burke–Ernzerhof (PBE) type [22] generalized-gradient-approximation (GGA) functional. The Heyd–Scuseria–Ernzerhof (HSE06) [23] hybrid exchange-correlation functional is adopted to correct the band gap problem. The 12×12×1 (6×6×1) k−grids with the energy cut-off of 400 eV are used in PBE (HSE) simulations. Atomic positions are optimized with the residual force less than 0.02 eV/Å. The Wannier wave-functions and tight-binding Hamiltonian are constructed from V−d and Se−p orbitals based on the DFT results using the vasp2wannier90 interface [24]. The band structures and edge states of $1T-V{\mathrm{Se}}_{2}$ ribbon are calculated using the semi-infinite Green function simulations with the Sancho–Rubio method [25].

To carry out the Wilson loop [26,27] and Hall conductivity, we start with the Berry phase ϕ [28] by

(1) $$\phi_{m}\left(k_{i}\right)=-\mathrm{im}\;\log\;\sum_{i}\langle k_{i}|k_{i+1}\rangle .$$

Here, $|k_{i}\rangle$ is the wave-function of the *m*th band at $k_{i}$ point. Then, the Berry curvature of the *m*th band, $\Omega_{m}\left(k\right)$, is calculated via the Stokes’ theorem

(2) $$\Omega_{m}\left(k\right)=\lim_{\delta a\to 0}\frac{\int \Omega_{m}\cdot da}{\delta a}=\lim_{\delta a\to 0}\frac{\phi_{m}\left(k,\delta a\right)}{\delta a}.$$

The intrinsic Hall conductivity $\sigma_{H}\left(E,T\right)$ of energy *E* and temperature *T* is just the Ω integral over Brillouin zone (BZ) [29], that is

(3) $$\sigma_{H}\left(E,T\right)=\frac{e^{2}}{2\pi \hbar }\int_{\mathrm{BZ}}f_{\mathrm{FD}}\left(E,T\right)\;\Omega \cdot d^{2}k.$$

Here $f_{\mathrm{FD}}\left(E,T\right)$ is the Fermi–Dirac distribution. The 600×600 k−grids are used for Hall conductivity calculations in this work.

## 3. Result and Discussion

**Figure 1:**
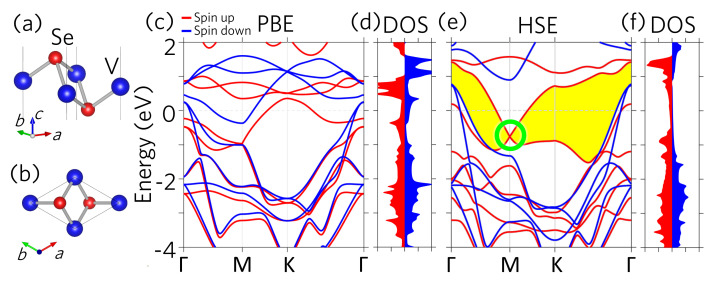
(**a**) Side-view and (**b**) top-view of the monolayer $1T-V{\mathrm{Se}}_{2}$ lattice structure. (**c**) Band structures given from PBE simulations. The red (blue) lines indicate the spin up (down) bands. (**d**) Density of states (DOS) from PBE. The red (blue) region presents the spin up (down) contributions. (**e**) Band structures and (**f**) DOS obtained using HSE functional. The green circle indicates the Weyl point (WP).

Figure 1a,b show the lattice structure of $1T-V{\mathrm{Se}}_{2}$ monolayer. Similar to other transition metal dichalcogenides in the 1T phase, the transition metal atom, vanadium (V), locates at the center plane sandwiched between two chalcogen anions, selenium (Se). By adopting the experimental lattice constant a=b= 3.36 Å [14,30], the optimized V–Se bond length is 2.49 Å with the corresponding interlayer distance being 1.56 Å.

Figure 1c,d illustrate the band structures and density of states (DOS) given from the PBE calculations, respectively. The electronic bands near the Fermi level (above −1 eV) are mainly contributed from V−d orbitals. Owing to the broken time-reversal symmetry, the spin up (red) and spin down (blue) bands are relatively shifted due to the Zeeman effect in the V−d orbitals. The exchange splitting around the Fermi level is roughly 1 eV, leading to the magnetic moment of 0.71 $\mu_{B}$ per V ion. As for the Se−p orbitals, which contributes mainly below −1 eV, the band splitting between the spin up and spin down channels are thus much weaker due to the trivial Zeeman effect therein.

The exchange-correlation potential and band gap are usually underestimated in DFT simulations [31], including the local-density approximation (LDA) and PBE functional. Although LDA (PBE) underestimates band gaps due to the self-interaction problem, the Hartree–Fock approach, on the other hand, overestimates band gaps because no correlation is considered. Therefore, the Heyd–Scuseria–Ernzerhof (HSE06) [23] hybrid exchange-correlation functional is usually adopted to correct the band gap problem by adding a portion of Hartree–Fock exact exchange functional to PBE (LDA). The HSE hybrid functional has been widely used in 2D material simulations [32,33,34,35] for obtaining correct results in comparison with experimental observations. Consequently, we also perform HSE calculations to avoid possible band gap problems in this work as discussed below.

Figure 1e,f show the HSE band structures and DOS, respectively. As expected, the band gaps are significantly enhanced by the HSE functional, especially around the Fermi level. In particular, there opens up a continuous band gap, as shown in the yellow region (Figure 1e). Accordingly, the DOS peaks of V−d orbitals are pushed away from the Fermi level, resulting in the enhanced magnetic moment of 1.1 $\mu_{B}$ per V ion. A remarkable emerging feature is the band crossing at the M point by two spin up single bands at the energy of −0.75 eV as highlighted by the green circle in Figure 1c. Some of the previous works have named such crossings in 2D systems as Weyl points [36,37,38], even though the Weyl point was originally defined in three-dimensional materials [39]. In this work, following previous works, we hereafter call this band crossing as the Weyl point. Our analysis as discussed below also shows band inversions around the Weyl point, implying that a topological phase occur in this TMD monolayer, similar to the two $p_{z}$ bands crossing at *K* points in graphene [2] and *X*ene monolayer [40].

**Figure 2:**
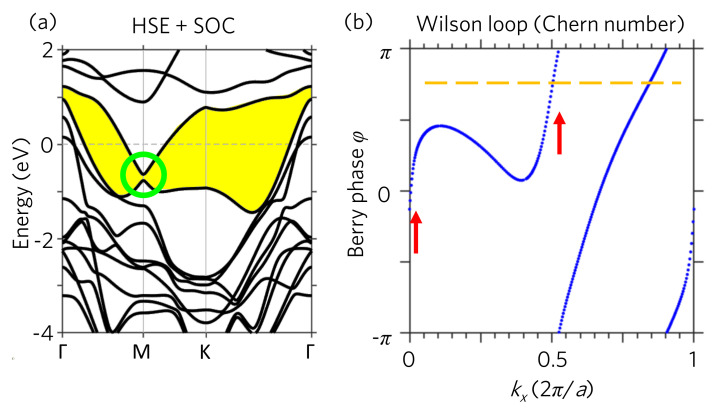
(**a**) HSE+SOC band structure of monolayer $1T-V{\mathrm{Se}}_{2}$. The yellow region presents the continuous band gap and the green circle indicates the SOC band gap. (**b**) Wilson loop of monolayer $1T-V{\mathrm{Se}}_{2}$ with HSE functional and SOC. The red arrows highlight the sharp slope in the Wilson loop. The two crossings through the reference line (the orange dashed line) indicate that the Chern number is two (C=2).

To study the topological phase of monolayer $1T-V{\mathrm{Se}}_{2}$, we include the spin–orbit coupling (SOC) in the self-consistent-field calculations using the HSE functional. As shown in Figure 2a, the SOC opens up an energy gap at the Weyl point, forming a complete continuous band gap (the yellow region) through out the Brillouin Zone (BZ). Consequently, the topological behavior can be well defined below this continuous band gap by performing the Wilson loop calculations. The Wilson loop [26,27] (also known as Wannier charge center, WCC [27]) simulations for Chern number are shown in Figure 2b. Different from the topological $Z_{2}$ index, which can only be 0 or 1, the Chern number of QAH effect can be any non-negative integer Z, such as 0, 1, 2, and 3, etc. As can be seen in Figure 2b, the Berry phase winds for two times when going through the first Brillouin zone, indicating that the Chern number is two (C=2). On the contrary, the Wilson loop simulations using PBE functional with SOC result in the Chern number C=0 as shown in Supporting Materials. This indicates that the PBE functional, which underestimate the band gap, gives a topologically trivial normal magnetic metal ground state for monolayer $1T-V{\mathrm{Se}}_{2}$. Further, the two sharp slopes in the Wilson loop simulations based on HSE results as highlighted by the red arrows in Figure 2b indicates that the Berry curvatures originate from the Γ and M points. Our Wilson loop simulations evidence that the monolayer $1T-V{\mathrm{Se}}_{2}$ is a QAH material with the topological invariant C=2, that is, two topologically protected edge states can be found at the layer boundary.

**Figure 3:**
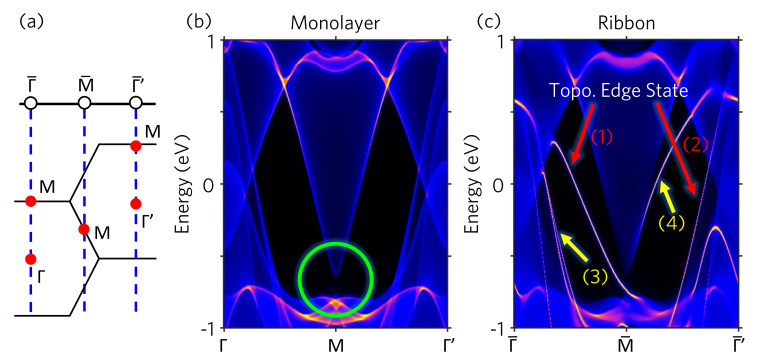
(**a**) 2D and 1D Brillouin zone of $1T-V{\mathrm{Se}}_{2}$ monolayer and ribbon at the (010) edge, respectively. The blue dash lines indicate the relation between the high symmetry k−points in the 2D and 1D BZ. (**b**) Band structure of $1T-V{\mathrm{Se}}_{2}$ monolayer from the semi-infinite Green functions method. (**c**) Band structure of $1T-V{\mathrm{Se}}_{2}$ ribbon from the semi-infinite Green functions method. In comparison with (**b**), four edge states (bright yellow curves) can be identified. Two of them are topological edge states as indicated by the red arrows (1) and (2). The other two edge states are topologically trivial as indicated by yellow arrows (3) and (4).

To show the topological edge states on the (010) boundary of the $1T-V{\mathrm{Se}}_{2}$ ribbon, we perform the semi-infinite Green function calculations. Figure 3a illustrates the relation between the 2D and 1D BZ of $1T-V{\mathrm{Se}}_{2}$ monolayer and (010) ribbon, respectively. The M point in the 2D BZ projects to the $\bar{M}$ point of the (010) 1D BZ. The Γ and M points project to the $\bar{\Gamma }$ point of the (010) 1D BZ. The band structure of the 2D monolayer is depicted in Figure 3b. The green circle in Figure 3b highlight the SOC band gap at the M point corresponding to that shown in Figure 2a. In comparison with the band structure of the 1D (010) ribbon shown in Figure 3c, one can clearly identify four edge states. Among them, two edge states indicated by red arrows (1) and (2) connecting the conduction and valence bands are topologically non-trivial. Whereas the other two edge states indicated by yellow arrows (3) and (4) are topologically trivial. The edge state (3), which is coincident with the 2D bulk band edge as compared with Figure 3b, can be identified as the edge resonance state. Although the edge state (4) connecting the conduction band at $\bar{M}$ to the conduction band at $\bar{\Gamma }$ is thus a topologically trivial edge state. Our Green-function simulations present two topological edge states in good consistency with our Wilson loop simulations discussed in the last paragraph.

To detect the edge states of two-dimensional materials is a great challenge in experiments. However, the Hall conductivity and spin Hall conductivity measurements serve as a much more viable approach for evidencing the edge states. The intrinsic Hall conductivity can be obtained from the Hall conductivity of spin up and down channels via

(4) $$\sigma_{H}=\sigma_{H}^{↑}+\sigma_{H}^{↓}.$$

Here the $\sigma_{H}^{↑}$ ($\sigma_{H}^{↓}$) is the intrinsic Hall conductivity of the spin up (down) channel, which can be calculated through the Berry curvature calculations as described in the Section 2. In addition, the intrinsic spin Hall conductivity is defined by

(5) $$\sigma_{H}^{\mathrm{spin}}=\sigma_{H}^{↑}-\sigma_{H}^{↓}.$$

In this work, we focus on the ferromagnetic phase of monolayer $1T-V{\mathrm{Se}}_{2}$, i.e., below the Curie temperature $T_{\mathrm{Curie}}=470\;K$ [14] while above the CDW phase transition at $T_{\mathrm{CDW}}=130\;K$ [14]. Therefore, we consider an intermediate temperature T=200K for the Fermi-Dirac distributions in Equation (3).

**Figure 4:**
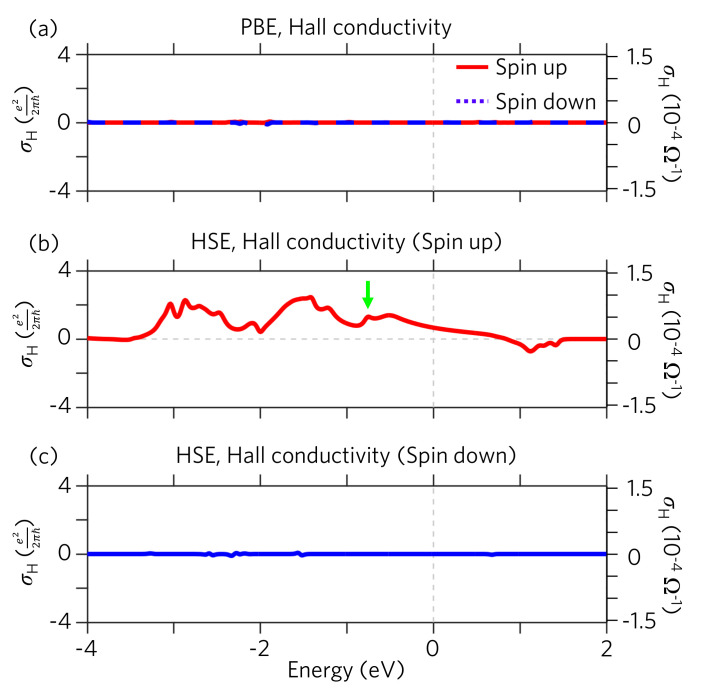
The intrinsic Hall conductivity from DFT simulations with temperature T=200K. (**a**) The Hall conductivity of spin up and down electrons from PBE simulations. The left (right) axis show the Hall conductivity in the unit $\frac{e^{2}}{2\pi \hbar }$ ($10^{-4}\;\Omega^{-1}$). (**b**,**c**) The Hall conductivity of spin up and down electrons from HSE simulations. The green arrow highlights the peak enhanced by the band crossing point.

The calculated intrinsic Hall conductivity of $1T-V{\mathrm{Se}}_{2}$ monolayer from PBE is presented in Figure 4a. The Hall conductivity of both the spin up and down channels are nearly zero, indicating the topological trivial phase given from PBE. This is consistent with the zero Chern number discussed in our previous Wilson loop calculation using PBE. As the HSE hybrid exchange-correlation potentials are taken into consideration, significant Hall conductivity emerges in the spin up channel owing to the topological non-trivial Chern number of C=2, as shown in Figure 4b. The Hall conductivity curve shows a peak value of 1.25 $\frac{e^{2}}{2\pi \hbar }$ (or $4.85\times 10^{-5}\;\Omega^{-1}$) at the band crossing energy, as indicated by green arrow in Figure 4b. It is worth noting that this value is not equal to two because the band splitting in $1T-V{\mathrm{Se}}_{2}$ happens within a continuous energy gap rather than within a full band gap. The Hall conductivity integral (Equation (3)) over the energy instead of over band numbers thus does not yield a corresponding integer number. As the energy increases, the Hall conductivity decreases, leaving a smaller value of 0.67 $\frac{e^{2}}{2\pi \hbar }$ (or $2.58\times 10^{-5}\;\Omega^{-1}$) at the Fermi level. On the other hand, the Hall conductivity of the spin down channel, which is topologically trivial, remains zero (Figure 4c). Based on the HSE results, we conclude the following relation for the Hall conductivity of $1T-V{\mathrm{Se}}_{2}$:

(6) $$\sigma_{H}≃\sigma_{H}^{\mathrm{spin}}≃\sigma_{H}^{↑}.$$

This relation presents a clear route to experimentally examine the topological properties of the 2D TMD material $V{\mathrm{Se}}_{2}$ in the 1T phase.

A large number of recent studies have demonstrated various classes of topological phases with magnetism both experimentally [9,10,11,12,41,42] and theoretically [43,44,45,46,47,48,49]. However, 2D monolayer materials with quantum anomalous Hall effect have not been shown by experiments to date. On the other hand, there have been several DFT simulations for $1H-V{\mathrm{Se}}_{2}$ and $1T-V{\mathrm{Se}}_{2}$ [50,51], but the topological phase has not been report yet. The Hall conductivity study on monolayer $1H-V{\mathrm{Se}}_{2}$ [52] shows that $\sigma_{H}≃0$, implying that $1H-V{\mathrm{Se}}_{2}$ is topologically trivial. Our results thus provide a timely interesting results for the topological phases in 2D TMD materials with magnetism, and thus invite future theoretical and experimental studies toward this direction.

## 4. Conclusions

In summary, we propose that $1T-V{\mathrm{Se}}_{2}$ monolayer is a quantum anomalous Hall (QAH) semimetal with the same Hall conductivity and spin Hall conductivity, i.e., the same charge Hall current and spin Hall current. The HSE hybrid exchange-correlation functional demonstrate a topological phase with Chern number C=2 in $1T-V{\mathrm{Se}}_{2}$ monolayer, resulting in a 2D QAH semimetal. We present 2 topological edge states for the $1T-V{\mathrm{Se}}_{2}$ ribbon, intrinsic Hall conductivity $\sigma_{H}$, and intrinsic spin Hall conductivity $\sigma_{H}^{\mathrm{spin}}$. We confirm that this intrinsic spin Hall conductivity is robust against the temperature variation for it appears in a wide range of several eV near the Fermi level (see Figure 4b). This indicates that $1T-V{\mathrm{Se}}_{2}$ is a good candidate for experimentally realizing 2D QAH at room temperature and, thus, is highly applicable in spintronics device based on topological and Hall properties [53,54,55]. Previous study has discovered the external-magnetic-field dependent conductivity, which implies anomalous Hall conductivity [15]. This is consistent with our finding, and thus $1T-V{\mathrm{Se}}_{2}$ could be a new kind of stoichiometric quantum anomalous Hall material. The intrinsic quantum anomalous hall effect has been reported in twisted bilayer graphene experiment with very low $T_{\mathrm{Curie}}≃7.5\;K$ [56]. Our study demonstrates that the anomalous Hall conductivity and spin anomalous Hall conductivity can be observed in $1T-V{\mathrm{Se}}_{2}$ with $T_{\mathrm{Curie}}≃470\;K$, which opens up a new route to room-temperature spintronics. Moreover, our study can also be extended to other two-dimensional magnetic materials, such as ${\mathrm{CrI}}_{3}$, ${\mathrm{CrGeTe}}_{3}$, and ${\mathrm{Fe}}_{3}{\mathrm{GeTe}}_{2}$.

## Data Availability

The data is available on reasonable request from the corresponding author.

## Supplementary Materials

The following are available online at https://www.mdpi.com/article/10.3390/nano11081998/s1. Figure S1: The band structures and the Wilson loop simulations of monolayer 1T-VSe2 with PBE exchange-correlation potential and spin-orbital coupling. (a) The band structure. The yellow region indicates continuous band gap. (b) The Wilson loop result, without crossing along the reference line (the orange dashed line), shown the Chern number C = 0 in PBE simulations; Figure S2: The Hall conductivity of monolayer 1T-VSe2 with different temperatures, from HSE result. (a) (b) Spin up and down channel with T = 150 K. (c) (d) Spin up and down channel with T = 300 K.
