# Supplementary material for: Topological Phase and Quantum Anomalous Hall Effect in Ferromagnetic Transition-Metal Dichalcogenides Monolayer 1T − VSe2
Source: Nanomaterials (Basel). 2021 Aug 4;11(8):1998. doi: 10.3390/nano11081998 (PMC8401610; doi:10.3390/nano11081998)
Supplement: Supplementary file 1 [file nanomaterials-11-01998-s001.zip › nanomaterials-1262630-supplementary.pdf]

Supporting Information

Topological phase and quantum anomalous Hall effect in ferromagnetic transition-metal dichalcogenides monolayer 1T-VSe2

Angus Huang,[1, 2] Chin-Hsuan Chen,[1] Ching-Hao Chang,[2] and Horng-Tay Jeng[1, 3, 4]

1. *Department of Physics, National Tsing Hua University, Hsinchu 30013, Taiwan*
2. *Department of Physics, National Cheng Kung University, Tainan 70101, Taiwan*
3. *Physics Division, National Center for Theoretical Sciences, Hsinchu 30013, Taiwan*
4. *Institute of Physics, Academia Sinica, Taipei 11529, Taiwan*

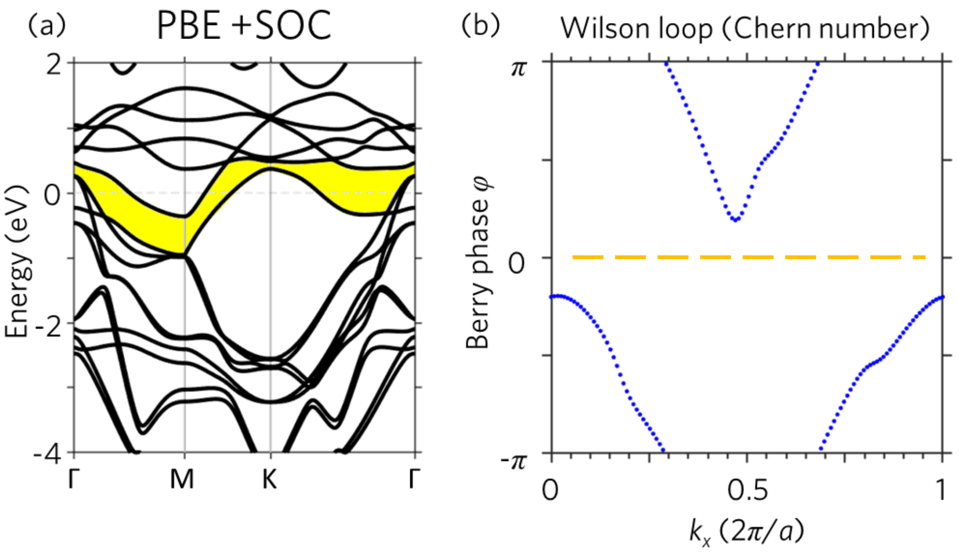

Figure S1. The band structures and the Wilson loop simulations of monolayer 1*T*-VSe_2 with PBE exchange-correlation potential and spin-orbital coupling. (a) The band structure. The yellow region indicates continuous band gap. (b) The Wilson loop result, without crossing along the reference line (the orange dashed line), shown the Chern number $C = 0$ in PBE simulations.

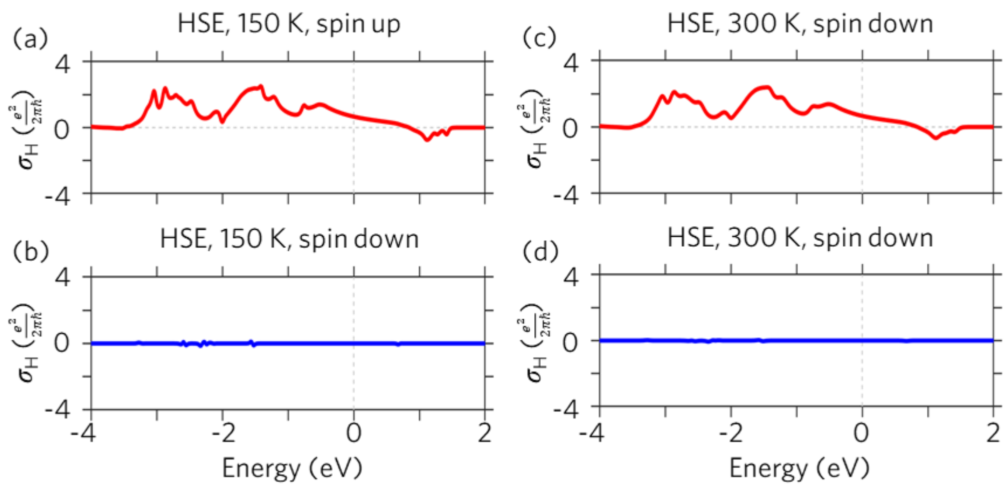

Figure S2. The Hall conductivity of monolayer 1*T*-VSe_2 with different temperatures, from HSE result. (a) (b) Spin up and down channel with $T = 150$ K. (c) (d) Spin up and down channel with $T = 300$ K.
